# The impact of healthcare reform on the efficiency of public county hospitals in China

**DOI:** 10.1186/s12913-017-2780-4

**Published:** 2017-12-20

**Authors:** Shuai Jiang, Rui Min, Peng-qian Fang

**Affiliations:** 1 School of Medicine and Health Management, Tongji Medical College, Huazhong University of Science and Technology, 13 Hangkong Road, Qiaokou District, Wuhan, Hubei 430030 China; 2 Academy of Health Policy and Management, Huazhong University of Science and Technology, 13 Hangkong Road, Qiaokou District, Wuhan, Hubei 430030 China

**Keywords:** Healthcare reform, County hospital efficiency, Data envelopment approach, China

## Abstract

**Background:**

The new round of Healthcare Reform in China has implemented over 3 years since 2009, and promoted greatly the development of public county hospitals. The purpose of this study is to evaluate county hospitals efficiency before and after the healthcare reform, and further assess the reform effectiveness through the comparative analysis of the efficiency.

**Methods:**

Data envelopment analysis (DEA) was employed to calculate the efficiency of 1105 sample hospitals which were selected from 31 provinces of China, also, Tobit regression was used to regress against those main external environmental factors.

**Results:**

Our results show that the scales and amounts of service of hospitals had increased sharply, however, the efficiency was relatively low and decreased slightly from 2008 to 2012. Thirteen (1.18%) in 2008 and six (0.54%) hospitals in 2012 were defined as technically efficient, and the average scores were 0.2916 and 0.2503. The technical efficiency average score of the post-reform was significantly less than that of the pre-reform (*p* < 0.001), and the score of eastern region was highest and the western was lowest among three regions of China.

**Conclusions:**

It suggests the reform had not well improved county hospital efficiency although hospitals have reached a fair developing scale, and the corresponding policies and measures should be put into effect for improving efficiency, especially in the level and structure of health investment, operation and supervision mechanism of county hospitals.

## Background

In March 2009, Chinese government formally launched the Healthcare Reform. Moreover, the government committed to spending an additional CNY 850 billion (USD 125 billion) in the ensuing 3 years for achieving comprehensive universal health coverage by 2020 [1]. Those core contents of the healthcare reform are focusing on the reform of wide range of medical insurance coverage, national essential drug system, medical care and public health service system, basic public health service and pilot reform of public hospitals [2]. One of the major tasks in the new round of healthcare reform is the public hospital reform, as we know, the main reason is that county hospital is leading role in the Rural Three-level Health Service Network, its service covers more than 900 million people of China, accounting for 70% of the whole population [3]. The content of public hospital reform includes substantial increases in public investment, restructuring of the hospital management system, and correction of the tendency for commercialization [2].

In the rural areas of China, the health service system, i.e. Rural Three-level Health Service Network, is made up of county and its subordinate health organizations, including government-run county hospital, township health center and village clinic, in which county hospital is the central of technical guidance and treatment in the system, township health center is the hinge of county and village health services, and village clinic is the base. They form health service tertiary structure aiming to meet the demand of grassroots level health services. Of which, county hospitals as health service providers in the healthcare system are important carriers for the government to provide basic medical and health services to county residents.

In China, hospitals include General Hospitals, Traditional Chinese Medicine Hospitals, Integrated Chinese and Western Medicine Hospitals, Traditional Ethnic Medicine Hospitals, Specialized Hospitals and Nursing Homes by category. On the other point of view, they are classified into three levels according to the number of hospital beds: tertiary level, secondary level and primary level. Tertiary hospitals have more than 500 beds, mainly treating complicated diseases and providing specialized medical care, technical guidance, medical education and scientific research. Secondary hospitals have 100–499 beds, providing comprehensive medical services and undertaking a part task in teaching and scientific research, while primary hospitals have 20–99 beds, providing common disease treatment and prevention, rehabilitation, and primary health care services. In the study, sample hospitals are county general hospitals. Generally, the vast majority of county hospitals are secondary hospitals.

As we all know, the area administered by the People’s Republic of China is divided to five level divisions: Provincial level (1st), Prefectural level (2nd), County level (3rd), Township level (4th) and Village level (5th). In addition, County level include Districts (under the Jurisdiction of Cities), County-level cities, Counties, Autonomous counties. In 2015, there were a total of 1875 counties (or county-level cities, autonomous counties) and 8951 county general hospitals in rural areas, according to China Statistics Yearbook (CSY) and China Health Statistics Yearbook (CHSY). Those counties covered all 31 provinces, autonomous regions, and municipalities (31 provinces, for short) of mainland China, which are distributed throughout the developed eastern China (Eastern China), moderately developed central China (Central China) and undeveloped western China (Western China).

Seen from different regions, the provinces are generally divided into three regions based on their geographical locations and socioeconomic status indicated by GDP per capita and average income. The eastern China region include (11 provinces): Beijing, Fujian, Guangdong, Hainan, Hebei, Jiangsu, Liaoning, Shandong, Shanghai, Tianjin, and Zhejiang; The central China region include (8 provinces): Anhui, Heilongjiang, Henan, Hubei, Hunan, Jiangxi, Jilin, and Shanxi; The western China region include (12 provinces): Chongqing, Gansu, Guangxi, Guizhou, Inner Mongolia, Ningxia, Qinghai, Shaanxi, Sichuan, Tibet, Xinjiang, and Yunnan.

According to the task scheduling of county hospital reform, governments at all levels have gradually increased their health input, but the imbalance in regional economic development impacted on capacity of government input, and further affected the development of county hospitals. The reform had been carried out for over 3 years, it was time for exploring hospital development status. During 2008–2012, the number of beds in county hospitals expanded from 1105.26 thousand to 1708.08 thousand, increased by 54.54%; medical personnel from 1378.35 thousand to 1858.42 thousand, increased by 34.83%; the total outpatient and emergency visits from 590.00 million to 866.95 million people, increased by 46.94%; and inpatients from 335.30 million to 599.29 million, increased by 78.78% [4]. This suggests that the scale of county hospitals has expanded and the number of visits and inpatients has improved largely since healthcare reform, therefore, it is an important issue to evaluate whether hospital performance has improved.

Hospital efficiency is one of the key indicators of hospital performance [5], and has been the significant subject of numerous health economics studies [6]. The efficiency study of county hospitals of 31 provinces in China was few according to literature reviews. Hu et al. [7, 8] carried out related studies on Chinese regional hospital efficiency and determinants of efficiency. However, most of these studies were only focused on efficiency of hospitals in unique province [9–14], and found that efficiency of public hospitals still need improvements. This paper focused on evaluating the efficiency of county hospitals in China before and after healthcare reform and exploring external determinants of hospital efficiency. The empirical study objectively evaluated the effect of China’s healthcare reform and provided constructive references for policy makers and hospital managers, besides, it would conduced to the international comparison of hospital efficiency.

## Methods

### Data source and study design

In our study, the sample hospital data were from the database of National Institute of Hospital Administration (NIHA) of National Health Family Planning Commission of PRC and the Provincial Statistical Yearbook issued by Provincial Statistical Bureau, which include the hospitals’ basic facility information, financial statements, health manpower, medical services quantity and quality from 2006 to 2012. The data in 2008 (pre-reform) and 2012 (post-reform) only were used, which were pre- and post-reform data to assessment the operational efficiency of county public hospitals.

**Fig. 1:**
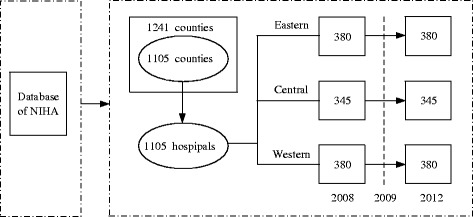
Study design of this paper

The design requires choosing one general hospital each county, and samples extracted from the database through setting rigorous retrieve fields in computer system. Eventually, 1241 county hospitals (from 1241 counties) were selected as the research samples. Considering availability and integrity of data, there were 1105 hospitals selected as the research objects, the research objects comprised 380, 345, and 380 hospitals from the eastern, central and western China regions. Data source and study design of this study shown in Fig.1.

### Efficiency evaluation methods

Data Evaluation Analysis (DEA), as a non-parametric linear programming technique, have been widely applied to measure hospital efficiency [11, 15–17]. However, conventional DEA approaches do not adjust the environmental effects and slacks while computing the efficiency of decision making units (DMUs) according to standard production theory, and the result could be seriously biased. To calculate corrected efficiency scores, a four-stage DEA model was proposed [18].

In this paper, the four-stage DEA is used to compute the constant returns to scale technical efficiency (TE_CRS_), variable returns to scale technical efficiency (TE_VRS_) and scale efficiency (SE) of county public hospitals. In the course of operation, an input-oriented DEA is employed in that the demand for health services cannot be controlled and health managers can determine only those resources attributed to each hospital to provide those services adequately [19]. In China, hospital manager, as we know, can ask for more resources (health human resource, medical infrastructures, hospital beds and buildings, government finance or subsidy, etc.) by applying to the county health bureau (or development & reform commission) under the county health development plan.

In the first stage, traditional inputs and outputs are calculated the efficiency and input slacks of DMUs on the basis of a standard input-oriented VRS DEA model without concerning socio-economic, environmental and other exogenous variables.

In the second stage, it is to explore the relationship between the total slacks (TS) obtained from the first stage as the dependent variable and exogenous variables as the independent variables, using Tobit regression analysis, which is good for left- or right-censored observations. Here, the regression equations are specified as:

$$ {\displaystyle \begin{array}{c}{TS}_{ij}={f}_i\left({E}_{ij},{\beta}_i\right)+{\mu}_{ij}\\ {}i=1,2,\dots \mathrm{m}.\kern0.5em j=1,2,\dots \mathrm{n}.\end{array}} $$

Where *TS*_*ij*_ represent the total slacks computed of the *i*-th input of the *j*-th DMU in the first stage, *E*_*ij*_ is a vector of exogenous variables, *β*_*i*_ is a vector of coefficients for exogenous variables, *u*_*ij*_ is the random disturbance term.

In the third stage, estimated coefficients from the regression are used to predict total slack for each input and for each unit based on its external environment factors. These predictions are used to adjust the primary input data for each unit according to the difference between maximum predicted slack and predicted slack. This creates a new pseudo data set where the inputs are adjusted for the influence of external conditions:

$$ {\displaystyle \begin{array}{c}{X}_{ij}^{adjust}={X}_{ij}^{original}+\left[\mathit{\operatorname{Max}}\left\{{TS}_{ij}\right\}-{TS}_{ij}\right]\\ {}i=1,2,\dots \mathrm{m}.\kern0.5em j=1,2,\dots \mathrm{n}.\end{array}} $$

In the fourth stage, it is to use the adjusted data set to re-run the DEA model again under the initial input-output specification and generate new measures of radial inefficiency. These radial scores measure the inefficiency that is attributable to management [19].

### Variables selection

Regarding input and output variables, it has no appropriate unified variables for DEA model so far, and in general, input and output variables are selected by the previous empirical research and international literature review [5, 20–27]. The inputs usually include three broad categories: labor (health human resources), materials (drugs, etc.) and capital (buildings and equipment, etc.) [28]. Variables selection generally follow the representativeness, measurement convenience and availability of data. In the paper, the number of physicians, nurses and medical technicians (people of pharmacy department, clinical laboratory, medical imaging department, radiology department and other medical auxiliary departments) and the number of actual open beds (as a proxy indicator for capital inputs) are selected as input variables, while the number of outpatient and emergency visits and inpatient days are selected as output variables. The use of “inpatient days” instead of “inpatients” or “discharge patients” is more medically homogeneous and preferably represents hospital output [9, 11]. The four input and two output variables are selected as shown in Table 1.

**Table 1:** Descriptive statistics of inputs and outputs variables (*N* = 1105)

Variables	2008		2012		2008–2012	
	Mean	S.D.	Mean	S.D.	t-value	*p-value*
Input variables						
Actual open beds	309	181	451	280	−31.298	0.000
Physicians	141	87	172	105	−18.026	0.000
Nurses	165	105	241	162	−26.314	0.000
Medical technicians	47	38	70	50	−16.945	0.000
Output variables						
Outpatient & emergency visits	167,058	147,222	243,824	222,001	−25.636	0.000
Inpatient Days	93,502	73,128	159,258	153,037	−18.226	0.000

In this study, we mainly explore external environment factors which are not controlled by the hospital managers or operator. The samples are all public county hospitals, which are not for profit and some relevant data was unavailable, so those characteristics like GDP per capita (*yuan*) (GDPPC), catchment population (10 *thousand persons*) (CPOP), proportion of government subsidy to hospital income (%) (GSUB) and the region where a hospital is situated (REG) are expected to affect the efficiency of hospitals. Here, we set two regional dummy variable REG_1 (if eastern =1 and other =0) and REG_2 (if western =1 and other =0) referring to the central.

The descriptive analysis of the input, output and exogenous variables was conducted using SPSS statistical software (version 16.0). Technical efficiency of county hospitals was calculated using DEAP analytical software (version 2.1). The Tobit regression analyses was performed with STATA statistical software (version 12.0).

## Results

The descriptive statistics (mean, standard deviation, minimum and maximum) for inputs and outputs variables are shown in Table 1. In 2008, the 1105 hospitals, using a total of 341,445 beds, 155,805 physicians, 182,325 nurses and 51,935 technicians, produced those outputs of 184,599.09 thousand outpatient and emergency visits and 103,319.71 inpatient days. In 2012, the 1105 hospitals serviced 269,425.52 thousand outpatient and emergency visits and 175,980.09 inpatient days. Those outputs were produced using a total of 498,355 beds, 190,060 physicians, 266,305 nurses and 77,350 technicians.

Parameter estimates are summarized in Table 2. Obviously models were statistically significant (*p* < 0.001). Catchment population had a significant positive coefficient in four inputs, respectively. However, the proportion of government subsidy to hospital income had a significant negative coefficient in four inputs, respectively. The central region were taken as the reference cases, the eastern region had a significant positive coefficient in physicians in 2008 and a significant negative coefficient in beds, nurses and technicians in 2012, and the western region also had a significant negative coefficient in four inputs in 2008 and in those three inputs except beds in 2012.

**Table 2:** Tobit regression coefficient of slacks for input variables (N = 1105)

Variables	2008				2012			
	beds	physicians	nurses	technicians	beds	physicians	nurses	technicians
GDPPC	0.0002	0.0001	0.0001	0.0000	0.0004^**^	0.0001^*^	0.0002	0.0000
CPOP	1.8263^**^	0.8290^**^	1.0680^**^	0.3135^**^	2.3414^**^	0.9364^**^	1.4324^**^	0.3076^**^
REG_1	10.6845	12.4863^**^	0.5774	−3.7579	−44.5729^**^	−4.0521	−34.8444^**^	−9.9991^**^
REG_2	−20.8811^**^	−30.7351^**^	−29.1477^**^	−16.3398^**^	−18.8853	−20.2540^**^	−24.9102^**^	−14.5810^**^
GSUB	−1.7946	−0.2280	−0.8977^**^	−0.2817^**^	−4.0388^**^	−0.8824^**^	−2.0503^**^	−0.5297^**^
Constant	113.7894^**^	50.4160^**^	66.5448^**^	28.5075^**^	201.1246^**^	71.1763^**^	117.3873	44.2452^**^
Log likelihood	−6457.3	−5855.4	−5887.0	−5182.0	−7015.2	−5999.5	−6496.5	−5416.5
LR chi2(10)	565.18^***^	432.45^***^	597.79^***^	266.03^***^	440.74^***^	426.79^***^	410.28^***^	217.05^***^
Pseudo R2	0.0419	0.0356	0.0483	0.0250	0.0305	0.0343	0.0306	0.0196

**Notes**: (a) *Significant at the 0.10 level, two-tailed test. **Significant at the 0.01 level, two-tailed test. *** Significant at the 0.001 level, two-tailed test. (b) GDPPC: GDP per capita. CPOP: catchment population. REG_1: dummy variable (if eastern =1 and other =0) and REG_2: dummy variable (if western =1 and other =0) referring to the central. GSUB: proportion of government subsidy to hospital income

County hospital efficiency scores in the pre-reform (in 2008) and post-reform (in 2012) are shown in Table 3. For TE_CRS_, the (Mean ± S.D.) scores were 0.2916 ± 0.1839 and 0.2503 ± 0.1717. Thirteen (1.18%) and Six (0.54%) hospitals were defined as technically efficient_,_ while the remaining 1092(98.82%) and 1099(99.46%) hospitals were inefficient. Among the latter, only 3.26% and 2.17% hospitals had an efficiency score of more than 0.750, and mostly 49.77% and 60.18% hospitals scored less than 0.249. In terms of TE_VRS_, the (Mean ± S.D.) scores were 0.6986 ± 0.0965 and 0.5934 ± 0.0998. Twenty-three (2.08%) and eleven (1.00%) hospitals were classified as pure technically efficient, while the remnant 1082(97.92%) and 1094(99.00%) hospitals operated inefficiently. The efficiency scores of 25.61% and 6.34% hospitals were more than 0.750, and 73.21% and 81.45% hospitals scored 0.500–0.749. With respect to SE, the (Mean ± S.D.) scores were 0.4214 ± 0.2458 and 0.4145 ± 0.2396. Twenty-three (2.08%) and six (0.54%) hospitals showed constant returns to scale (CRS), meaning they operated at their most productive scale. 1069(96.74%) and 1080(97.74%) hospitals revealed increase returns to scale (IRS), suggesting that their scale should be expanded to reach scale efficient. Thirteen (1.18%) and nineteen (1.72%) hospitals experienced decrease returns to scale (DRS), implying they should scale down to become scale efficient.

**Table 3:** Description and pairwise tests for hospital efficiency scores in stage four between 2008 and 2012 (*N* = 1105)

Regions	Efficiency	2008	2012	Z-value	*p-value*	Efficiency ranking	*N* (%)	
							2008	2012
All	TE_CRS_							
	Mean	0.2916	0.2503	−16.291	0.000	100%	13(1.18%)	6(0.54%)
	S.D.	0.1839	0.1717			75.0–99.9%	23(2.08%)	18(1.63%)
	Min	0.020	0.006			50.0–74.9%	94(8.51%)	66(5.97%)
	Max	1.000	1.000			25.0–49.9%	425(38.46%)	350(31.67%)
	Skew(SE)	1.328(0.074)	1.559(0.074)			0–24.9%	550(49.77%)	665(60.18%)
	TE_VRS_							
	Mean	0.6986	0.5934	−24.671	0.000	100%	23(2.08%)	11(1.00%)
	S.D.	0.0965	0.0998			75.0–99.9%	260(23.53%)	59(5.34%)
	Min	0.441	0.296			50.0–74.9%	809(73.21%)	900(81.45%)
	Max	1.000	1.000			25.0–49.9%	13(1.18%)	135(12.22%)
	Skew(SE)	0.671(0.074)	1.146(0.074)			0–24.9%	0	0
	SE							
	Mean	0.4214	0.4145	−1.797	0.072	100%	21(1.90%)	6(0.54%)
	S.D.	0.2458	0.2396			75.0–99.9%	120(10.86%)	109(9.86%)
	Min	0.026	0.011			50.0–74.9%	220(19.91%)	245(22.17%)
	Max	1.000	1.000			25.0–49.9%	435(39.37%)	419(37.92%)
	Skew(SE)	0.642(0.074)	0.626(0.074)			0–24.9%	309(27.96%)	326(29.50%)
Eastern	TE_CRS_	0.3874	0.3443	−8.248	0.000			
	TE_VRS_	0.7030	0.6122	−12.611	0.000			
	SE	0.5531	0.5494	−1.038	0.299			
Central	TE_CRS_	0.2704	0.2231	−10.438	0.000			
	TE_VRS_	0.7059	0.5979	−14.164	0.000			
	SE	0.3850	0.3729	−1.567	0.117			
Western	TE_CRS_	0.2151	0.1809	−9.782	0.000			
	TE_VRS_	0.6876	0.5706	−15.911	0.000			
	SE	0.3227	0.3172	−0.410	0.682			

A Wilcoxon signed-rank test showed that the technical efficiency average score of the post-reform was significantly less than that of the pre-reform, and was statistically significant (*p* < 0.001), and the scale efficiency average score of the post-reform is less than that of the pre-reform but was statistically insignificant at the 5% level of significance (*p* = 0.072).

**Fig. 2:**
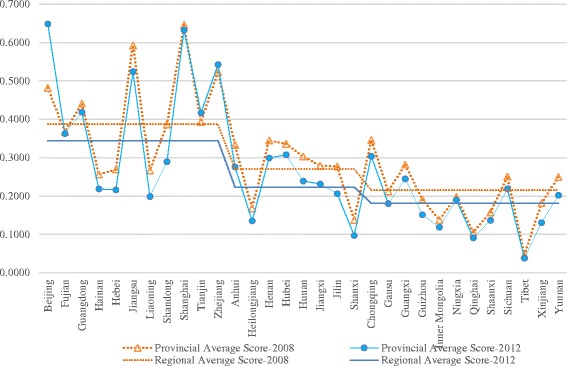
Technical efficiency average scores of hospitals by province in stage four between 2008 and 2012

Figure 2 intuitively demonstrated the technical efficiency average score for each province (or region) between pre- and post-reform. Scores of the other 28 provinces except for Beijing, Tianjin and Zhejiang were decreased from pre- to post-reform, and different provinces had wide different hospital efficiency scores, and the score difference of different provinces within eastern was bigger than that within the other two regions.

Table 3 indicated hospital efficiency average scores in different regions between pre- and post-reform, and the score of eastern region was highest and the western was lowest among those regions. A Wilcoxon signed-rank test showed that the technical efficiency (TE_CRS_ and TE_VRS_) average score of each region in post-reform was statistically lower than that in pre-reform (*p* < 0.001), while the scale efficiency average score of each region in post-reform was less than that in pre-reform but was statistically insignificant (*p >* 0.100).

## Discussion

In this study, the comparative analysis of hospital efficiency in pre- and post-reform revealed the effectiveness of China’s healthcare reform. However, some external environment factors except for the reform itself affected hospital efficiency. As can be seen from the results above, counties with more catchment population made hospitals experience more slacks of inputs, the reason was that more people accessing to medical services resulted in county hospitals increase unreasonably their inputs to meet medical service demands, such as beds and health personnel. In China, government subsidy, which relies on joint funding by central and local governments, was mainly used for constructing hospital infrastructure, purchasing advanced equipment and improving medical staff salary. Thus sufficient subsidy was conducive to improve hospitals service ability and efficiency without hiring more medical personnel. Compared with the central region, the eastern and western hospitals markedly decreased the input slacks in post-reform, suggesting that it was less excess use of input than the central. It is mainly due to the fact that hospital managers put emphasis on optimizing the medical staff structure, improving medical technology and operating capability.

The efficiency average score of hospitals went down after controlling those external environment factors, it manifests that the hospital efficiency is greatly affected by external environment, and some confounding factors lead to the increased pseudo-efficiency. Seen from the fourth stage, the technical efficiency average score of technical efficiency went down from 0.2916 in 2008 (pre-reform) to 0.2503 in 2012 (post-reform), and scores of 60.18% hospitals were below 24.5% in 2012. Given the results, these hospitals can provide the same current level of outputs using 29.16% and 25.03% of their resources and without increasing inputs and only with good and wise management and the effort of employees. It was suggested that the healthcare reform toward Chinese county hospitals seems not to better have improved hospital technical efficiency.

From the regional perspective, the efficiency presented the tendency of the highest score in Eastern and the lowest score in Western China, suggesting that the characteristic “Geographical Advantages” produced by China’s policy orientation promotes high quality medical resources and advanced governance concepts and systems to be used preferentially in relatively developed eastern region. However, the orientation would result in the inequity of health resource allocation, which were huge inequalities across regions and between urban and rural areas [29]. The eastern was more inequitable than the central and western region, and the internal differences in the eastern region were relatively larger than other regions [30], so the score difference within eastern was bigger. To improve equity in resource allocation, more resources will be targeted to lower-income regions and rural areas [31]. Some scholars study on the modern hospital management system of county public hospitals and put forward the four kinds of ‘corporate governance model’, which are the internal management mechanism reform, the separation of supervision and operation, the separation of administrative units and institutions and the type of hospital ownership change, to the improvement of hospital governance and management [3, 32].

In addition, the SE average scores of 67.42% hospitals were lower than 0.500, while the TE_VRS_ scores of 87.78% hospitals were higher than 0.500, suggesting the number of technically efficient hospitals was more than that of scale efficient hospitals. Meanwhile, the TE_VRS_ average score was higher than SE score, it was implied that the low TE_CRS_ mainly attributed to the low SE. Under the reform, Chinese county hospitals have been experiencing an expansion in infrastructure and health workforce, especially in beds and workers, but the blind and unreasonable expansion, which local government is mostly responsible for funding regardless of the purpose and utilization efficiency of funds, led to the hospital invalid input and low scale efficiency. For Beijing, Tianjin and Zhejiang, their increased TE_CRS_ average scores after the reform resulted from the size of the hospital being better controlled to enhance hospital scale efficiency, as developed areas, their TE_VRS_ being improved significantly.

### Limitation

This study has several limitations needed to be mentioned. For one thing, our evaluation of county public hospitals just for about 3 year after the healthcare reform, to some extent, led to reducing the stability and robustness of the evaluating results, and the study design still needed to be optimized. For another, the selected variables were not perfect, especially, some input variables might be included in the DEA model, such as equipment, medical cost or expenses. Besides, the county hospital efficiency may be affected by some other environmental factors such as the structure of the population (% of older - 65 years and above, % of young 0–15 years), average length of stay (ALOS), Herfindahl-Hirschman Index (HHI, describes market competition for hospitals). It require us to do a further track research for county hospital operating efficiency in the future and alternative methods are encouraged to be applied for evaluating the hospital efficiency. However, despite its limitations, this study can be considered as a useful preliminary study towards exploring the county hospital efficiency by the four-stage DEA model.

## Conclusions

This study is a comprehensive nationwide study that assesses the Chinese county hospital efficiency before and after the healthcare reform. The average efficiency of 1105 hospitals was relatively low and decreased slightly from pre- to post-reform, it suggests that healthcare reform had not well improved county hospital efficiency. The hospital development imbalance caused the differences of hospital efficiency in different regions, namely the eastern hospital efficiency was better than the central and western. Therefore, some relative support policies and measures should be issued to optimize regional health resources allocation and raise medical technology and service ability to improve hospital efficiency.

The regression analysis on examining external environment factors of slack for each input revealed that when one county’s population was large so that existing hospitals could not provide adequate service for them, it should construct a new hospital or support the relatively weak hospital become better one rather than blindly expanse existing hospitals’ scale. It also suggests that the efficiency evaluation, as a dynamic management strategy in the process of healthcare reform, should be carried out annually for timely adjustment of hospital development strategy.

## Abbreviations

CRS: Constant returns to scale
CSY: China Statistics Yearbook
DEA: Data envelopment analysis
DMUs: Decision making units
NIHA: National Institute of Hospital Administration
S.D.: Standard deviation
SE: Scale efficiency
TE_CRS_: Constant returns to scale technical efficiency
TE_VRS_: Variable returns to scale technical efficiency
VRS: Variable returns to scale
